# Oxidative Stress as a Risk Factor for Hearing Changes in HIV-positive Normal Listeners

**DOI:** 10.6061/clinics/2020/e1845

**Published:** 2020-11-10

**Authors:** Carla G. Matas, Fernanda Yasmin OMM Padilha, Rosanna MG Angrisani, Alessandra G. Samelli

**Affiliations:** Curso de Fonoaudiologia, Faculdade de Medicina (FMUSP), Universidade de Sao Paulo, Sao Paulo, SP, BR

**Keywords:** HIV, Hearing, Hearing Disorders, Oxidative Stress, Hearing Tests

## Abstract

**OBJECTIVES:**

Human immunodeficiency virus-positive (HIV+) individuals can experience a decrease in antioxidants. Such deficiency can make inner ear cells and synapses more vulnerable to oxidative stress, resulting in auditory alterations, even in the presence of normal thresholds. This study aims to compare the audiological findings of HIV+ patients (with and without exposure to anti-retroviral treatment) to those of healthy individuals.

**METHODS:**

This was a cross-sectional observational study, comprising 42 normal-hearing adults divided into the Control Group (CG), without HIV; Group I (GI), HIV+, without exposure to the highly active anti-retroviral therapy (HAART); Group II (GII), HIV+, with exposure to HAART. All participants underwent conventional audiometry (0.25-8 kHz), high-frequency audiometry (9-20 kHz), transient evoked otoacoustic emissions (TEOAEs), efferent auditory pathway’s inhibitory effect assessment, brainstem auditory evoked potentials (BAEPs), and cognitive potential (P300).

**RESULTS:**

In the comparison of the hearing thresholds between the groups, there was a statistically significant difference for most of the frequencies assessed (GII presented hearing thresholds significantly poor when compared with other groups). The presence of TEOAE and the inhibitory effect was also verified in a significantly higher number of individuals in the CG than in the other groups. As for the BAEP, there was a statistically significant difference for the interpeak intervals I-V (GII showed higher values when compared with CG). For P300, there were no statistically significant differences.

**CONCLUSION:**

Normal-hearing HIV+ individuals (with and without exposure to HAART) presented with poor performance in the audiological procedures, suggesting the presence of auditory alterations even in the presence of normal-hearing thresholds.

## INTRODUCTION

Acquired immunodeficiency syndrome (AIDS) is caused by the human immunodeficiency virus (HIV), a specific type of retrovirus that affects the immune system, possibly leading to various opportunistic infections and potentially affecting the central nervous system (1).

Many HIV-infected individuals present with a preserved level of immune cells, remaining asymptomatic for long periods, not clinically manifesting the disease. According to the Brazilian Ministry of Health (2), HIV-seropositive individuals are considered to have AIDS when their CD4+T lymphocyte count is lower than 350 cells/mm^3^.

Currently, the most commonly used therapy in infected individuals is the highly active anti-retroviral therapy (HAART), which involves the use of at least three drugs and monitoring the viral plasma concentration (3).

Hearing loss in HIV-positive individuals can be caused by various factors, including direct effects from HIV, increased susceptibility to opportunistic infections in the middle ear and central nervous system, and treatment with potentially ototoxic medications (4,5).

Reactive oxygen species (ROS) are oxygen mediators with a high reactive capacity for many biological molecules. ROS are produced during various cellular processes and in many organelles (6).

In certain pathological conditions or when several medications are being taken, the exaggerated production of ROS takes place, leading to what is known as oxidative stress. Oxidative stress is caused by the imbalance between ROS production and its removal through strategies and antioxidants available in the organism, including enzymes, proteins, molecules, and vitamins (7).

In individuals with HIV, the depletion of antioxidants essential for the maintenance of the electrolytic balance of the immune system cells can occur, causing a decrease in immune response, and consequently, an increase in HIV replication, possibly aggravating the patient’s condition (8,9).

A previous study has shown that individuals with HIV have increased levels of biomarkers of oxidative damage to deoxyribonucleic acid (DNA) in the CD4+T cells, as well as decreased DNA glycosylase activity for the repair of oxidative-based lesions in those cells (10).

Studies have shown that oxidative stress contributes to the death of hair cells, which can lead to auditory alterations (11,12). Recently, the oxidative stress resulting from noise exposure (13-15) and ototoxic medications (4,16) are being related to auditory alterations, even in the presence of hearing thresholds within normality (16-18). This condition is called hidden hearing loss, which can be characterized by the presence of auditory complaints (tinnitus, hyperacusis, reduced speech-in-noise intelligibility) in individuals with normal-hearing thresholds (19-21). Thus, these possible auditory alterations would be verified through other tests, in addition to conventional audiometry.

Hence, it is important that different conditions (for instance, the presence of HIV and/or the use of anti-retroviral medications) that generate oxidative stress be evaluated, for understanding the influence of these factors on the auditory system to advance.

This study aims to compare the audiological findings of HIV+ patients (with and without exposure to anti-retroviral treatment) to those of healthy individuals, all of whom had hearing thresholds within normality.

## MATERIAL AND METHODS

This was a cross-sectional observational study, approved by the Ethics Committee for the Analysis of Research Projects (CAPPesq - Medical School Clinics Hospital of the University of São Paulo - FMUSP) under the number 1026/04. All the participants signed the informed consent form in compliance with the Brazilian National Health Council Resolution 466/12.

The sample comprised 42 individuals with normal-hearing (hearing threshold <25 dB HL in the frequencies of 0.25 to 8 kHz) (22), with an age range of 25-52 years. The individuals were divided into three groups with their respective inclusion criteria:

- Group I (GI), comprising 13 HIV-positive individuals (verified through serology) without exposure to anti-retroviral treatment;
- Group II (GII), comprising 14 HIV-positive individuals (verified through serology) exposed to anti-retroviral treatment (combined therapy or HAART, which consists of at least three of the following medications: lamivudine, zidovudine, efavirenz, didanosine, nevirapine, lopinavir, tenofovir, stavudine, indinavir, abacavir, amprenavir, ritonavir, and atazanavir);
- Control Group (CG), comprising 15 individuals without HIV (evaluation report and seronegative); audiological, language, or auditory processing complaints; and any history of psychiatric or neurological diseases.

The following exclusion criteria were considered for the three above-mentioned groups: pure-tone audiometry with altered hearing thresholds, presence of active opportunistic infections, presence of clinical and/or cognitive impairment preventing or hindering audiological and/or electrophysiological examinations to be performed, and history of otologic surgery and/or otologic diseases unrelated to HIV.

GI and GII were referred by the House of AIDS - Zerbini Foundation and by the municipal system health services specialized in sexually transmitted diseases (STD/AIDS) of the São Paulo Municipal Department of Health. The CG comprised a convenience sample.

### Procedures

The medical reports of the individuals from GI and GII were analyzed; their otologic history was verified through anamnesis.

The audiological assessment included the visual inspection of the external acoustic meatus; acoustic immittance measures (tympanometry and acoustic reflexes) to verify the condition of the middle ear; conventional air-conduction pure-tone threshold audiometry at the frequencies of 0.25 to 8 kHz; and air-conduction high-frequency audiometry at 9 to 20 kHz (Grason-Stadler GSI 61, TDH-50 earphones) in an acoustically treated room, with standard audiometric techniques.

Subsequently, for the transient evoked otoacoustic emissions (TEOAEs) to be picked up with and without contralateral noise, the ILO 92 (Otodynamics) system was used. The stimulus was a nonlinear click with an intensity ranging from 78-84 dB SPL peak. For the TEOAE, the responses were considered present when the signal-to-noise ratio was greater than 3 dB SPL in all frequency bands (23). In the cases where the TEOAEs were present, the efferent auditory pathway’s inhibitory effect was calculated by subtracting the TEOAE amplitudes in the presence of noise (white noise to the contralateral ear at 60 dB SPL) from the TEOAE amplitudes in the absence of noise. Differences resulting in positive values were considered the presence of an efferent auditory pathway’s inhibitory effect (24).

To perform the electrophysiological assessment (brainstem auditory evoked potential [BAEP] and cognitive potential [P300]), the Express Traveler Portable System, from Bio-Logic, was used, along with THD-39 earphones.

For the BAEP, the click stimulus with rarefied polarity was used, presented monaurally at 80 dB HL, at a presenting speed of 19.3/s, lasting 0.1 milliseconds, totaling 2,048 stimuli. The absolute latency values of waves I, III, and V, as well as interpeak intervals I-III, III-V, I-V, were analyzed using the normality standard proposed by the Evoked Potential User Manual of Bio-Logic.

To pick up the P300, the tone-burst stimulus was used, presented monaurally at 75 dB HL, at a presenting speed of 1.1 stimulus per second, totaling 300 stimuli. The frequent stimulus (80% - 240 stimuli) was presented at 1,000 Hz, and the rare stimulus (20% - 60 stimuli) at 1,500 Hz. The individuals were instructed to remain alert and pay attention to the rare stimuli, which appeared randomly among a series of frequent stimuli (oddball paradigm), mentally counting whenever they appeared (thus performing the cognitive activity). In the trace resulting from the subtraction of the rare stimulus from the frequent stimulus, the latency of the P300 component was analyzed. As normality criteria, the values proposed by McPherson (25) were considered for P300 latency waves.

### Statistical analysis

Descriptive analyses and hypotheses tests were conducted. The one-factor Analysis of variance (ANOVA), Tukey’s, and chi-square tests were used. Initially, the left and right ears of each group were compared for each procedure. As no significant differences were found, the right and left ears were grouped together and then compared between the groups. A *p*-value<0.05 was considered significant.

## RESULTS

Regarding the hearing thresholds, statistically significant differences were verified between the groups for all frequencies assessed in the conventional and high-frequency audiometry, except for 9 kHz. In general, it was noted that individuals in the CG presented better hearing thresholds compared with those in GI and GII, and in turn, the GI’s hearing thresholds were better than those of GII (Tables 1 and 2).

Table 3 shows the distribution of individuals by the TEOAE results (presence or absence), as well as of the efferent auditory pathway’s inhibitory effect. It was verified that the CG presented 100% present responses for TEOAE, while the other two groups presented approximately 50%. As for the inhibitory effect, with a statistically significant difference, the CG showed more present responses when compared with the other two groups.

Concerning the P300 and BAEP latencies, the three groups presented similar values (Table 4). The only statistically significant difference was observed for interpeak I-V, in which GII’s values were higher when compared with the CG.

Table 5 shows the distribution of normal or altered results of individuals by each electrophysiological procedure. For BAEP, when compared with GI and GII, there was a significantly higher number of normal results in CG. GII presented a higher alteration percentage when compared with GI and CG. For P300, there were no statistically significant differences between the groups.

## DISCUSSION

Oxidative stress occurs when ROSs reach high levels, which cannot be neutralized by the defense mechanisms, thus damaging and altering the functions of biological molecules, potentially harming the cells. These alterations can be observed in the presence of pathological conditions, as in the case of AIDS, and when various medications are being taken, as, for instance, anti-retrovirals (6,26). In the auditory system, oxidative stress can make cells more vulnerable, contributing to the death of cells and alterations in the auditory pathway (11,12).

The frequent manifestation of auditory abnormalities, from the middle ear to the central auditory nervous system, in individuals with HIV/AIDS has already been known for some time (27). Nevertheless, some recent studies suggest that people with oxidative stress can refer to auditory complaints and present some alterations throughout the auditory system, even in the absence of abnormalities in pure-tone threshold audiometry (28,29).

Thus, this study sought to verify the occurrence of auditory alterations in normal-hearing HIV-positive individuals (with and without exposure to anti-retroviral treatment) comparing them with normal-hearing individuals without the virus.

The findings in this study highlighted that the study groups GI and GII presented hearing thresholds significantly higher in relation to the CG, although still within normality standards in pure-tone threshold audiometry. Similarly, high-frequency audiometry showed higher hearing thresholds than the other two groups. These results suggest that there are mechanisms of HIV and of the ototoxic medications (especially in GII) (30-34) that are harmful to the cochlea, which can be related to oxidative stress; this was observed in a study of noise-exposed individuals with an increase in high-frequency thresholds, precociously indicating the existence of damage to the cochlea, even in the presence of hearing thresholds within normality in conventional audiometry (35).

Recent studies in noise-exposed animals suggest that, in addition to the vulnerability of cochlear hair cells because of oxidative stress, there may concomitantly be a process that would damage the synapsis between the hair cells and auditory nerve fibers. This alteration in the synapses could cause an alteration in the processing of acoustic information, even in the presence of hearing thresholds within normality, contributing to the presence of abnormalities, such as speech-in-noise difficulties and/or tinnitus (11-13,36).

In the case of seropositive individuals participating in this study, the hearing thresholds of the conventional audiometry were within normality, while the absence of TEOAE was verified in approximately 50% of the individuals in GI and GII, which points to the existence of subclinical cochlear damage. Previous studies conducted in normal-hearing noise-exposed adults showed the absence of otoacoustic emissions for both types of evoked otoacoustic emissions (37,38). Thus, the absence or decrease in the amplitude of the otoacoustic emissions can provide the first indications of cochlear damage (39-41).

Regarding the efferent auditory pathway’s inhibitory effect, it was believed that the role of the olivocochlear efferent system was to protect the hair cells from noise exposure. However, after the vulnerability of the synapses between the hair cells and auditory nerve was discovered, some studies highlighted the efferent system’s protective role against synaptopathy. Two studies on guinea pigs exposed to noise (42) and age effect (43), respectively verified that the section of the efferent bundle exacerbated synaptopathy (44).

Concerning our findings, only half of the individuals belonging to GI and GII could be assessed for the inhibitory effect since this can only be measured in individuals who had TEOAE present. Of these, more than 70% of the individuals in GII and 100% of those in GI had no inhibitory effect. As the number of individuals with absent responses differed by only two between the two groups among seven individuals, it was challenging to make assumptions about the possible differences between GI and GII. However, the percentage of the presence of the inhibitory effect in CG differed significantly in relation to GI and GII.

Accordingly, analyzing the absence of the efferent auditory pathway’s inhibitory effect in more than 70% of the individuals in GI and GII, it can be suggested that these individuals present auditory alterations not restricted only to the cochlea. This is in agreement with the study by Carvallo et al. (45), which emphasized that the decrease or absence of the efferent system’s inhibitory effect can be a risk marker for auditory alterations.

For the results concerning BAEP, this study agrees with previous findings suggesting that HIV-seropositive individuals have a higher percentage of alterations, indicating an impairment of the central auditory pathway, regardless of exposure to the anti-retroviral treatment (46,47). However, regarding the localization of the alteration, Matas et al. (46) verified that the alteration of the lower brainstem was the most common; however, in this study, the groups with HIV showed statistically significant differences in the interpeak interval I-V, denoting diffused alteration in the brainstem, even for hearing within normality.

A previous study detected high levels of oxidative stress markers in the central nervous system of HIV-infected humans (48), which could explain the presence of more BAEP alterations in the HIV groups when compared with the CG, and even more alterations in the individuals exposed to anti-retroviral medications.

Concerning the P300 latency values in this study, these were similarities between groups, suggesting that individuals with HIV positive, with and without exposure to anti-retroviral treatment, did not present cognitive difficulties, behaving similar to the CG. This fact can be explained by the phase of AIDS the patients were in, as none of them were in the advanced stage of the disease when cognitive decline could occur. A previous study conducted by Fein et al. (49) emphasized that the P300 wave delay can be associated with the progression of the disease when cognitive impairment occurs. Another study demonstrated that in 20% of HIV-infected individuals, the first apparent symptom is neurological (50), with manifestations that can include auditory processing (51). Thus, future studies are needed to monitor the evolution of P300 responses in patients with HIV at different stages of the disease as well as to include tests that specifically assess cognitive function.

Oxidative stress has been shown to be an important factor in the pathogenesis of many diseases, including neurodegenerative diseases and AIDS. The increase in oxidative damage linked to mitochondrial DNA dysfunction contributes to aging, affecting different cell signaling systems and neuronal connectivity, potentially leading to the death of hair cells and neurons, as both are vulnerable to oxidative stress (11,2,27).

A literature review conducted by Jong et al. (27) highlighted that HIV could affect the auditory system entirely, similar to what occurs in aging. The authors emphasized that synaptopathy could be present in individuals with HIV, even before cochlear damage. Therefore, it is fundamental to perform auditory function assessments of all individuals diagnosed with HIV, including other procedures besides conventional audiometry, such as the otoacoustic emissions assessment, the electrophysiological assessment, and the speech-in-noise test.

Hence, the findings in this study suggest that the alterations observed throughout the auditory pathway (and not identified through conventional audiometry) can be related to oxidative stress caused by HIV itself and the anti-retroviral medications (in the case of GII). Therefore, the need for new studies to be developed on the subject is emphasized, as well as the use of complementary examinations, in assessing individuals with HIV, for the early detection of possible auditory alterations.

## CONCLUSION

Normal-hearing HIV-positive individuals, with and without exposure to anti-retroviral treatment, presented with poor performance in the behavioral, electroacoustic, and electrophysiologic audiological procedures, when compared with the CG, indicating the presence of auditory alterations even with hearing thresholds within normality. These findings suggest that oxidative stress can be an influencing factor in these alterations.

This study was carried out at the Speech Therapy and Audiology Research Laboratory in the Auditory Evoked Potentials of the Speech Therapy Course, in the Department of Physiotherapy, Speech Therapy and Audiology, and Occupational Therapy, Medical School, University of São Paulo.

## AUTHOR CONTRIBUTIONS

Matas CG and Samelli AG designed, provided interpretative analysis, analyzed data, wrote, and critically revised the paper. Padilha FY and Angrisani RM provided interpretative analysis, wrote, and critically revised the paper.

## Figures and Tables

**Table 1:** Mean and standard deviation (in dB HL) of the auditory thresholds from 0.25 to 8 kHz of both the ears, by group

	250 Hz	500 Hz	1,000 Hz	2,000 Hz	3,000 Hz	4,000 Hz	6,000 Hz	8,000 Hz
Groups	Mean (SD)	Mean (SD)	Mean (SD)	Mean (SD)	Mean (SD)	Mean (SD)	Mean (SD)	Mean (SD)
GI (n=26)	9 (7.3)	8 (6.4)	6.5 (5.2)	3.8 (5.8)	6.7 (7.2)	9.4 (6)	12.8 (6.8)	11.5 (8.8)
GII (n=28)	12.5 (6.7)	10.5 (6.2)	8.5 (5.2)	8 (6.7)	10 (6.6)	10.8 (8.7)	13 (9.1)	13 (6.8)
CG (n=30)	6.7 (4)	4.8 (3.8)	5.1 (4.2)	4 (4.4)	4 (4.6)	6 (5.7)	6 (6.7)	6.5 (6)
*p*-value	0.002*	0.001*	0.035*	0.011*	0.002*	0.027*	<0.001*	0.002*
Tukey’s test	GI=GII	GI=GII	GI=GII	GI≠GII	GI=GII	GI=GII	GI=GII	GI=GII
	GI=CG	GI=CG	GI=CG	GI=CG	GI=CG	GI=CG	GI≠CG	GI≠CG
	GII≠CG	GII≠CG	GII≠CG	GII≠CG	GII≠CG	GII≠CG	GII≠CG	GII≠CG

ANOVA test.

* *p*-value<0.05; SD: Standard deviation; GI: Group I; GII: Group II; CG: Control Group.

**Table 2:** Mean and standard deviation (in dB HL) of the auditory thresholds from 9 to 20 kHz of both the ears, by group.

	9 kHz	10 kHz	11.2 kHz	12.5 kHz	14 kHz	16 kHz	18 kHz	20 kHz
Groups	Mean (SD)	Mean (SD)	Mean (SD)	Mean (SD)	Mean (SD)	Mean (SD)	Mean (SD)	Mean (SD)
GI (n=26)	13.9 (10.2)	11.6 (11.4)	16.6 (15.1)	24.3 (24.9)	29.7 (26.2)	28.7 (22.7)	18.7 (12.1)	6.8 (7)
GII (n=28)	16.5 (9.9)	17 (9.3)	24.7 (11.3)	28.1 (18.2)	42.7 (19.4)	48.1 (9.3)	31.1 (5.5)	10.9 (6.2)
CG (n=30)	11 (8.5)	8.8 (9.1)	10.5 (11.6)	14.3 (14.7)	18 (17.1)	21 (15.8)	14 (10)	2.6 (3.8)
*p*-value	0.115	0.017*	0.001*	0.033*	<0.001*	<0.001*	<0.001*	<0.001*
Tukey’s test	-	GI=GII	GI=GII	GI=GII	GI=GII	GI≠GII	GI≠GII	GI=GII
		GI=CG	GI=CG	GI=CG	GI=CG	GI=CG	GI=CG	GI≠CG
		GII≠CG	GII≠CG	GII≠CG	GII≠CG	GII≠CG	GII≠CG	GII≠CG

ANOVA test.

* *p*-value<0.05; SD: Standard deviation; GI: Group I; GII: Group II; CG: Control Group.

**Table 3:** Distribution of the TEOAE results (presence or absence) and efferent auditory pathway’s inhibitory effect results, by individual (number and percentage).

Groups	TEOAE		Inhibitory effect	
	Present n (%)	Absent n (%)	Present n (%)	Absent n (%)
GI (n=13)	7 (53.8%)	6 (46.2%)	0 (0%)	7 (100%)
GII (n=14)	7 (50%)	7 (50%)	2 (28.6%)	5 (71.4%)
CG (n=15)	15 (100%)	0 (0%)	10 (66.6%)	5 (33.4%)
*p*-value	0.003*		0.005*	

Chi-square test.

* *p*-value<0.05; GI: Group I; GII: Group II; CG: Control Group.

**Table 4:** Mean and standard deviation (in ms) of waves I, III, and V; interpeak intervals I-III, III-V, and I-V; and P300 wave latencies of both the ears, by group.

	Wave I	Wave III	Wave V	Interpeak I-III	Interpeak I-V	Interpeak III-V	P300
Groups	Mean (SD)	Mean (SD)	Mean (SD)	Mean (SD)	Mean (SD)	Mean (SD)	Mean (SD)
GI (n=26)	1.62 (0.12)	3.79 (0.22)	5.78 (0.21)	2.15 (0.16)	4.16 (0.17)	1.99 (0.11)	337.4 (32.98)
GII (n=28)	1.60 (0.14)	3.78 (0.19)	5.81 (0.25)	2.18 (0.17)	4.21 (0.22)	2.02 (0.20)	323.21 (38)
CG (n=30)	1.57 (0.09)	3.74 (0.13)	5.69 (0.15)	2.16 (0.11)	3.96 (0.58)	2.1 (0.59)	317.8 (33.6)
*p*-value	0.374	0.596	0.108	0.819	0.040*	0.532	0.108
Tukey’s test	-	-	-	-	GI=GII	-	-
					GI=CG		
					GII≠CG		

ANOVA test.

* *p*-value<0.05; SD: Standard deviation; GI: Group I; GII: Group II; CG: Control Group.

**Table 5:** Distribution of electrophysiological assessment (BAEP, P300) results (normal or altered), by individual (number and percentage).

	BAEP		P300	
	Normal n (%)	Altered n (%)	Normal n (%)	Altered n (%)
GI (n=13)	8 (61.5%)	5 (38.5%)	10 (77%)	3 (23%)
GII (n=14)	6 (42.8%)	8 (57.2%)	14 (100%)	0 (0%)
CG (n=15)	14 (93.3%)	1 (0.7%)	14 (93.3%)	1 (0.7%)
*p*-value	0.014*		0.111	

Chi-square test.

* *p*-value<0.05; GI: Group I; GII: Group II; CG: Control Group.
